# Artificial intelligence in medical education curriculum: An e-Delphi study for competencies

**DOI:** 10.1371/journal.pone.0271872

**Published:** 2022-07-21

**Authors:** S. Ayhan Çalışkan, Kadir Demir, Ozan Karaca

**Affiliations:** 1 Department of Medical Education, Ege University Faculty of Medicine, Izmir, Türkiye; 2 Department of Management Information Systems, Izmir Democracy University Faculty of Economics and Administrative Sciences, Izmir, Türkiye; King Saud University, SAUDI ARABIA

## Abstract

**Background:**

Artificial intelligence (AI) has affected our day-to-day in a great extent. Healthcare industry is one of the mainstream fields among those and produced a noticeable change in treatment and education. Medical students must comprehend well why AI technologies mediate and frame their decisions on medical issues. Formalizing of instruction on AI concepts can facilitate learners to grasp AI outcomes in association with their sensory perceptions and thinking in the dynamic and ambiguous reality of daily medical practice. The purpose of this study is to provide consensus on the competencies required by medical graduates to be ready for artificial intelligence technologies and possible applications in medicine and reporting the results.

**Materials and methods:**

A three-round e-Delphi survey was conducted between February 2020 and November 2020. The Delphi panel accorporated experts from different backgrounds; (i) healthcare professionals/ academicians; (ii) computer and data science professionals/ academics; (iii) law and ethics professionals/ academics; and (iv) medical students. Round 1 in the Delphi survey began with exploratory open-ended questions. Responses received in the first round evaluated and refined to a 27-item questionnaire which then sent to the experts to be rated using a 7-point Likert type scale (1: Strongly Disagree—7: Strongly Agree). Similar to the second round, the participants repeated their assessments in the third round by using the second-round analysis. The agreement level and strength of the consensus was decided based on third phase results. Median scores was used to calculate the agreement level and the interquartile range (IQR) was used for determining the strength of the consensus.

**Results:**

Among 128 invitees, a total of 94 agreed to become members of the expert panel. Of them 75 (79.8%) completed the Round 1 questionnaire, 69/75 (92.0%) completed the Round 2 and 60/69 (87.0%) responded to the Round 3. There was a strong agreement on the 23 items and weak agreement on the 4 items.

**Conclusions:**

This study has provided a consensus list of the competencies required by the medical graduates to be ready for AI implications that would bring new perspectives to medical education curricula. The unique feature of the current research is providing a guiding role in integrating AI into curriculum processes, syllabus content and training of medical students.

## Introduction

The advent of artificial intelligence (AI) has reformulated, redefined and affected our day-to-day life in one or another manner including our professional careers to a great extent. AI in the healthcare industry has created tremendous interest in recent years, and healthcare trends are experiencing a noticeable shift in education and treatment [1, 2]. With recent advancements in digitized data collection, machine learning, and computational technology, AI applications are now permeating hastily into fields traditionally considered to be the human experts’ province [3]. So, the ‘*AI age*’ that contributes in creating technologies endowed with human-specific cognitive mechanisms is also expected to have a significant impact on the global healthcare system. Huge data collection, processing, and interpretation via Electronic Medical Records (EMRs) offers great potential benefits for healthcare services [2].

Considering the existing healthcare requirements and innovations in view, it can be conjectured that virtually every clinician will utilize AI technology for different purposes in the near future [4]. Artificial intelligence systems are predicted not to substitute doctors, but to perform certain jobs of doctors to bring them to a decent condition and to build a large variety of different fields of work [5]. The strongly impacted fields from the use of AI technologies in medical applications are image-based diagnosis for radiology, dermatology, ophthalmology, and pathology, genome interpretation, machine learning for biomarker discovery, inferring health status through wearable devices, autonomous robotic surgery, clinical outcome prediction and patient monitoring [3]. Hence, it is important for medical teachers to understand and recognize the potential of AI and prepare themselves to educate future doctors about these novel concepts [2, 6].

Physicians are in the early stages of a significant new task, primarily defined by capturing and entering patient data into EMR and AI systems [5], and parallel to this, medical education confronts a pedagogical challenge; how can AI systems in medicine be optimally incorporated into the curriculum to support the near and far future new physician roles [7]. According to Yu et al. [3], physicians’ roles need to be further upgraded as information integrators, consultants, and patient supporters to their existing positions, and the medical education system needs to equipp them by providing methodical training with desired AI techniques and methods to do so. In a recent study, Masters [5] looked in depth at how new physicians should become AI savvy; being proactive in AI system design, being able to work with AI diagnostic systems, interact with AI based systems, go deeper into consulting, where AI systems are not yet implemented optimally, train new medical AI applications and develop mental acquaintance with these new roles for better *AI age* adaptation.

Learners must comprehend well why AI technologies mediate and frame their decisions on medical issues. Formalization in terms of instruction on AI concepts can enable learners to grasp AI outcomes in association with their own sensory perceptions and thinking in the dynamic and ambiguous reality of daily medical practice. Besides, working cohesively with AI for physicians is a way to re-shape professional identity [7]. Furthermore, if the fundamental skills of machine learning methods are applied efficiently to next-generation physicians’ medical schools, it can enable them to become part of the medical artificial intelligence movement. Lately, a study performed by Pinto et al. [8] reports that medical students are cognizant of the potential of artificial intelligence systems and they show interest in its inclusion in the existing medical education. At this point, with the adoption of the effect of artificial intelligence transformation on health and education, it is expected that institutions providing medical education will include AI technology-based products in their education programs [9].

Keeping the potential of AI in view, our research question is “What are the competencies required by medical graduates to be ready for AI technologies in medicine and what is the expert panel’s consensus levels on those competencies?”

## Materials and methods

### The Delphi method

*The Delphi method* is a consensus agreement between the expert panelists through repeated iterations of anonymous opinions and proposed consensus statements by the researchers [10–13]. The Delphi process can involve a number of rounds before a consensus is attained among experts [11]. The responses to each Delphi round should be analysed by the researchers and reported to the respondents [14]. Adopting this process inadvertently encourages those with minority views among the experts to present their independent opinions [11].

The Delphi technique can be used in an academic medical environment to determine the weight of certain topics in the medical school curriculum [14].

Delphi studies have been conducted under online mode by many researchers in the past in diverse fields as it is both time and cost effective [14, 15]. This study was carried out using the online survey method to meet various experts safely and quickly during the time of the COVID-19 pandemic.

### Research design

**Fig 1:**
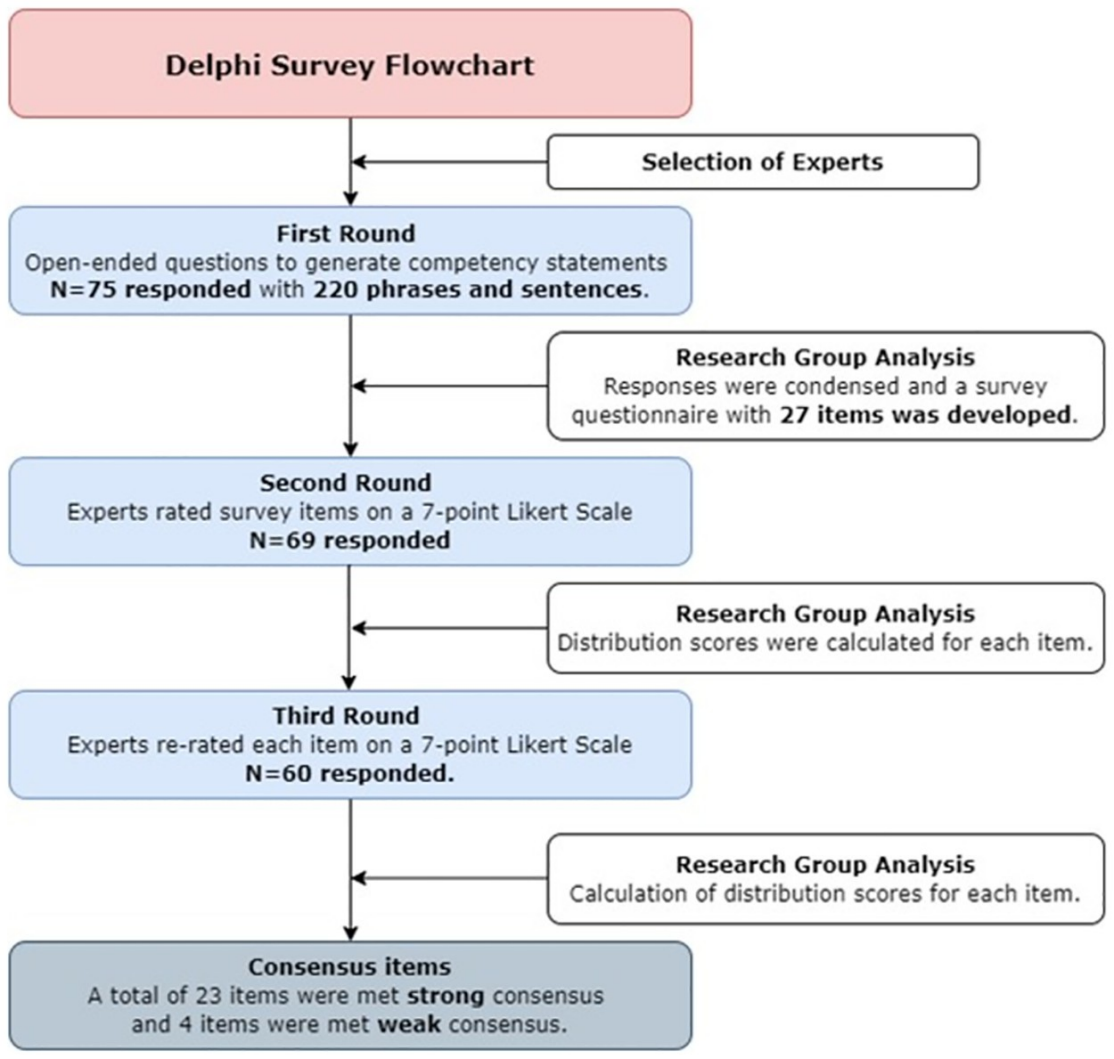
Delphi study diagram.

This study is an e-Delphi study—a form of classical Delphi, which was conducted via online survey questionnaires [16]. Ethical approval was granted by Ege University Scientific Research, and Publication Ethics Boards dated 28 November 2019 Ref.452. The participation of students was completely voluntary and written informed consent was obtained from all participants or, if participants are under 18, from a parent and/or legal guardian. The data collection took place between February 2020 and November 2020 using a free survey tool (LimeSurvey, limesurvey.org). Herein, Round 1 in the Delphi survey began with exploratory open-ended questions that invited and encouraged participants to provide their views on the topic [15]. Responses received in the first round were evaluated and refined to a 27-item questionnaire, which then sent to the experts to be rated using a 7-point Likert type scale. Similar to the second round, the participants repeated their assessments in the third round by using the second-round analysis. Following the analysis, it was decided that consensus had been reached after two rounds, such as similar studies [17, 18]. The process of performed Delphi study is shown in Fig 1.

### Selection of experts

The Delphi method does not use a random sample representing the target population but instead employs “experts” as panel members. In this study, “expert” was defined as academicians or practitioners either using or developing AI in healthcare. To ensure broad participation coverage, a scientific literature search was conducted to reach experts who are involved with AI technologies in healthcare. An invitation email was then sent to those experts to participate in the study, and in addition, a purposeful snowball sampling method was initiated with their assistance. A total of 128 Turkish experts from different institutes were reached and invited to participate from different backgrounds; (i) healthcare professionals/ academicians; (ii) computer and data science professionals/ academics; (iii) law and ethics professionals/ academics; and (iv) medical students were included in the initial Delphi round.

### Data collection and analysis

#### First round

A qualitative approach with open-ended questions was adopted for the first round. An online questionnaire in Turkish language was sent to the expert panel. They were asked to list all the competencies required by medical graduates to be ready for artificial intelligence technologies and possible applications in medicine. The questionnaire also included demographic information from the expert panel; age, gender, training, professions that they work in and academic title. Three reminders were sent to non-responders on a weekly basis via email to ensure the timely completion of the tasks. The participants responded with competency ideas in phrases or sentences. These contents were reviewed and analysed by the researchers. Same or similar phrases combined, resulting condensed items. The disagreements or discrepancies on any item/statement among or between researchers were resolved by open discussion to reach a final consensus.

#### Second round

An online survey questionnaire was sent to the expert panel who responded to the first round. The questionnaire comprised of 27 items, wherein the experts were asked to rate how much they agreed or disagreed with each of the items developed in the first round on a 7-point Likert scale (1: Strongly Disagree—7: Strongly Agree). Non-respondent participants received three reminders on a weekly basis. The researchers analysed the ratings, the distribution of ratings was established, and mean & median values were calculated.

#### Third round

**Fig 2:**
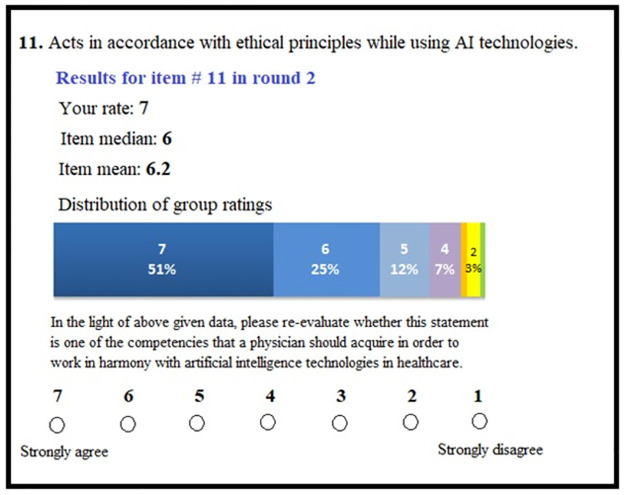
Example item presentation in Round 3 survey questionnaire.

The experts, who responded in the second round were given an individual online questionnaire comprising the same 27 items. In addition to own ratings in the second round, mean and median values and the distribution of the ratings were provided in the questionnaire and participants were asked to re-rate the items. One item’s representation in round 3 survey is exemplified in Fig 2. Three reminders were sent to non-responders on a weekly basis.

#### Consensus definition

Third round finalised the Delphi exercise. The agreement level and strength of the consensus was decided based on third phase results. Median scores were used to calculate the agreement level and interquartile range (IQR) were used for determining the strength of the consensus. IQR is the absolute value of the difference between the 75th and 25th percentiles with smaller values indicating higher degrees of the consensus. Interquartile range of 0 specifies a strong group consensus and 2 indicates dispersed responses. Responses, where the median was greater than or equal to 6 (high level of agreement) with a small IQR (less than or equal to 2) were considered that had reached strong consensus. Those with a median score less than or equal to 5, with a small IQR (less than or equal to 2), were considered to attain weak consensus [19].

#### Ethical considerations

The anonymity maintained during the entire Delphi study prevented experts from being exposed to peer pressure and encouraged them to put across their original ideas without being influenced by the ideas of others [13, 20]. The response rate of 70% was maintained during the study as it guaranteed both publication ethics and the anonymity feature of the Delphi technique [21]. In order to comply with this criterion, researchers know the identity of experts, but experts do not know each other.

#### Participation

Among 128 invitees, a total of 94 agreed to become members of the expert panel. Of them 75 (79.8%) completed the Round 1 questionnaire, 69/75 (92.0%) completed the Round 2 and 60/69 (87.0%) responded to the Round 3. The demographic characteristics of the participants are given in Table 1. Majority (≥ 87.0%) of the expert panel have advanced degrees and these characteristics remain similar across various rounds. Furthermore, a great proportion (≥ 84.1%) of the participants have advanced academic titles in which most of them were professors. The participants reflected a wide range of occupations both in medical and non-medical fields. Moreover, similar representation of the participants found within the occupation groups across different rounds.

**Table 1:** Demographic characteristics of participants in Delphi rounds.

Characteristics	Round 1 (N = 75)		Round 2 (N = 69)		Round 3 (N = 60)	
	N	%	N	%	N	%
Gender						
Female	19	25.3	18	26.1	16	26.7
Male	56	74.7	51	73.9	44	73.3
Age	45.2 (SD 9.8) years		45.2 (SD 10.0) years		44.8 (SD 9.9) years	
Education						
Undergraduate^a^	3	4.0	3	4.4	3	5.0
Bachelor	1	1.3	1	1.4	1	1.7
Master	5	6.7	5	7.2	3	5.0
PhD	66	88.0	60	87.0	53	88.3
Academic title						
Professor	33	43.4	30	43.5	26	43.3
Associate Professor	15	19.7	13	18.8	13	21.7
Assistant Professor	9	11.9	7	10.1	4	6.7
Research Assistant	8	10.5	8	11.6	8	13.3
None	11	14.5	11	15.9	9	15.0
Occupation^b^						
Medical	43	57.3	39	56.5	35	58.3
Non-medical	32	42.7	30	43.5	25	41.7

^a^Medical Student,

^b^Complete list is provided in S1 Appendix.

## Results

### Round 1

The participants submitted 220 competency phrases or sentences. After the content analysis, the same or similar statements combined, and a 41-item initial competency list was prepared. This initial list was sent to one medical academic professional and one computer & data science academic professional who were involved in using and developing AI tools/techniques in healthcare and their qualitative opinions were requested. Through the systematic review, combining items, omitting items, and wording changes were suggested. These suggestions were incorporated into the list and a 27-item competency list was obtained.

### Round 2

According to the expert panel response, median values of all items were greater than or equal to 5 with a maximum IQR value of 2. There were high levels of agreement for 23 items with medians in the strong range of agreement (6–7 on the seven-point Likert scale). The strength of agreement was high for all 23 items (IQR ≤ 2), which meant strong consensus achieved. The remaining 4 items revealed weak agreement level with median value of 5, resulting in a weak consensus (Table 2).

**Table 2:** Expert consensus on the competency items in the Delphi study.

Competency item	Round 2		Round 3	
	Median	IQR*	Median	IQR*
**Strong consensus**				
1. Uses health data in accordance with legal and ethical norms.	7.0	1.0	7.0	0.0
2. Acts in accordance with ethical principles while using AI technologies.	7.0	1.0	7.0	0.0
3. Uses artificial intelligence applications in accordance with its purpose.	7.0	1.0	7.0	1.0
4. Keeps the healthcare records in accordance with AI can process.	7.0	1.0	7.0	1.0
5. Uses information based on AI applications in combination with professional knowledge.	6.0	1.0	7.0	1.0
6. Values the use of AI for education, service and research purposes.	7.0	1.0	7.0	1.0
7. Explains how AI applications in healthcare offer a solution to which problem.	6.0	2.0	6.0	0.0
8. Organizes workflow in accordance with the working logic of AI.	6.0	2.0	6.0	0.0
9. Uses AI technologies effectively and efficiently in healthcare delivery.	6.0	2.0	6.0	0.25
10. Defines the basic concepts of data science	6.0	2.0	6.0	1.0
11. Follows the current developments and the literature regarding the use of AI technologies in healthcare.	6.0	1.0	6.0	1.0
12. Works as a team member with field experts in development process of AI applications.	6.0	2.0	6.0	1.0
13. Accesses, evaluates, uses, shares information and creates new information by using information and communication technologies.	6.0	2.0	6.0	1.0
14. Follows the legal regulations regarding the use of AI technologies in healthcare.	6.0	2.0	6.0	1.0
15. Expresses the importance of data collection, analysis, evaluation and safety; for the development of AI applications in healthcare.	6.0	2.0	6.0	1.0
16. Foresees the opportunities and threats that AI technology can create.	6.0	2.0	6.0	1.0
17. Differentiates between the functions and features of AI related tools and applications.	6.0	1.0	6.0	1.0
18. Properly analyzes the data obtained by AI in healthcare.	6.0	2.0	6.0	1.0
19. Defines the basic concepts and terminology of AI	6.0	1.0	6.0	1.0
20. Defines the basic concepts of statistics	6.0	2.0	6.0	2.0
21. Decides the use of AI technologies in healthcare.	6.0	2.0	6.0	2.0
22. Explains how physician knowledge and experience can be used in development of AI applications.	6.0	2.0	6.0	2.0
23. Chooses the proper AI application for the problem encountered in healthcare.	6.0	2.0	6.0	2.0
**Weak consensus**				
24. Explains how AI systems are trained	5.0	1.0	5.0	1.0
25. Explains the limitations of AI technology.	5.0	1.0	5.0	1.0
26. Explains the strengths and weaknesses of AI technology.	5.0	2.0	5.0	1.0
27. Explains the AI applications used in healthcare services to the patient.	5.0	2.0	5.0	2.0

*Interquartile range.

### Round 3

Consistent with round 2, there was a strong agreement on exactly the same 23 items and weak agreement on the remaining four (Table 2). One item’s median level increased from 6.0 to 7.0 and other median values of all items remained equal compared to round 2. In this final round, 12 items’ (item# 1,2,7,8,9,10,12,13,14,15,16,18) IQR level increased and 15 (item# 3,4,5,6,11,17,19,20,21,22,23,24,25,26,27) remained the same. Based on round 3 results, 23 items had reached strong consensus and four items had reached weak consensus (Table 2).

## Discussion and conclusion

This is the first Delphi study wherein a panel of experts from a spectrum of specialty fields reached consensus on the competencies required by medical graduates to be ready to utilize AI technologies and possible applications in medicine. Another distinctive feature of this research is the involvement of a relatively high number of participants throughout the study, right from the beginning until the completion of the study. Although, there are few Delphi studies employed with large groups, the median is reported as 17 participants [22]. Whereas, in this study, the expert panel had a high response rate, which remained proximate across groups and rounds, adding robustness to the findings.

In the establishment of structures that have not yet been created, the Delphi technique works very well to achieve a reliable result. It should be noted that the consensus achieved with the Delphi technique does not provide an accurate response, opinion, or decision, and only helps in defining areas that the group of experts considers to be important in relation to a particular topic [23, 24].

Another major strength of this study is the diverse composition of the expert panel. The panel members represented clinical and non-clinical perspectives in balanced proportions, which were supported by law and ethics field experts’ opinions. The diversity remained in clinical and non-clinical groups. As this study’s objective was mainly related to the learners’ perspective, i.e., the representation of students’ views in the study, which also enhances the robustness of the current findings. In addition, it was assured that an expert participating in this Delphi study must have knowledge and experience in the relevant field, and his/her opinions are respected by his colleagues. In fact, the constitution of the definition of expert provides a major contribution to the validity of this Delphi study [25].

The demographic data collected during this study showed a relatively advanced level of education among panel experts. The majority of the participants had PhD or medical residency degree and a great proportion had academic titles, with over 40% were professors. This ensured a well-established indicator of the expert panel’s field expertise and we argue that the responses based on these characteristics positively contributed to the results.

The unique feature of the current research that fills the gap present in the literature by providing a guiding role in integrating AI into curriculum processes, syllabus content [7] and physician training [2, 3, 6].

This study was conducted among the Turkish participants, including experts who have been educated and operated in Europe and North America. Furthermore, the participants’ relatively advanced level of education has brought a wider insight into the work and enhanced the possibility that its outcomes will be exploited and applied to medical schools worldwide.

Secondly, the rounds were held at intervals of four weeks, as performed in previous similar studies [18, 26, 27] and the total study was completed in seven months. In fact, the Delphi technique does not demand specific meeting times, which allow participants to make their decisions when they are ready [24]. Observing the time intervals suggested in the literature between Delphi tours contributed to both the validity and reliability of the study. However, the fact that the data collection process coincided with the ongoing Covid-19 pandemic period, wherein healthcare professionals work intensively, can be seen as a limitation.

Besides these limitations, ethical responsibility, anonymity, reliability, and validity concerns were maintained carefully throughout the data collection process [23, 24].

In education, curriculum development is a continuous process. In order to remain useful and germane, the curriculum must be responsive to changing values and expectations in education [28]. A number of researchers foresee a necessity for AI related new possible roles for doctors and deliberate changes in medical curriculum content that will be required to meet these new roles [5, 29, 30].

To respond to the current developments in AI technologies used in healthcare, specific intended achievements, in other words, competencies, have to be defined. This study has provided a consensus list of the competencies required by medical graduates to be ready for AI implications that would bring new perspectives to medical education curricula. Based on the current findings, it can be speculated that the competency list will contribute to the curriculum development process of medical schools and institutions.

The research designed to test and develop the items relied upon by an expert panel would be beneficial. In addition, it is proposed that these items should be used to develop the curriculum, to create syllabus content, and to conduct research on medical education. In addition, it is suggested that the results of this study should be further tested in different countries with a greater number of participants in order to explore the divergent perspectives in future studies.

With this study, the necessary physician competencies that can comprehend the working principles of medical AI, discuss its ethical aspects, and provide effective health services that are compatible with it, have been explored with the consensus of an expert panel. Therefore, the competence statements obtained in this study can be used as a guiding theoretical framework for medical education institutions of all levels that want to train physicians integrated with medical AI or to establish professional development programs for physicians in the healthcare field.

## Supporting information

S1 Appendix Occupation fields of the participants. (PDF)
