# Supplementary material for: Artificial intelligence in medical education curriculum: An e-Delphi study for competencies
Source: PLoS One. 2022 Jul 21;17(7):e0271872. doi: 10.1371/journal.pone.0271872 (PMC9302857; doi:10.1371/journal.pone.0271872)
Supplement: S1 Appendix — (PDF) [file pone.0271872.s001.pdf]

S1 Appendix

Occupation fields of the participants

Occupation	Round 1	Round 2	Round 3
		N (%)	
**Medical**	43 (57.3)	39 (56.5)	35 (58.3)
Anesthesiology	1	1	-
Biochemistry	3	3	3
Dentist	1	1	1
Emergency Medicine	1	1	1
Family Medicine	2	2	2
Health Administration	2	2	2
Health Informatics	1	1	1
Medical Education	2	2	2
Medical Ethics	2	2	2
Medical Genetics	2	2	2
Medical Student	3	3	3
Neuroscience	2	2	2
Neurosurgery	1	-	-
Occupational Medicine	1	1	-
Orthopedics	1	1	1
Pediatric Endocrinology	1	1	1
Pediatric Surgery	1	1	1
Public Health	1	1	1
Pulmonology	1	1	1
Radiology	9	7	5
Surgery	4	3	3
Urology	1	1	1
**Non-medical**	32 (42.7)	30 (43.5)	25 (41.7)
Bioinformatics	1	1	-
Biomedical Engineering	1	1	-
Computer Engineering	10	10	10
Econometrics	1	1	1
Educational Technologist	3	3	3
Electrical Engineering and Electronics	8	6	5
Electronics and Communication Engineering	1	1	1
Finance	1	1	-
Informatics	3	3	2
Law	2	2	2
Medical Engineering	1	1	1
**Total**	**75**	**69**	**60**
